# The Microstructures, Mechanical Properties, and Energetic Characteristics of a Novel Dual-Phase Ti_40_Zr_40_W_10_Mo_10_ High-Entropy Alloy

**DOI:** 10.3390/ma18020366

**Published:** 2025-01-15

**Authors:** Yuxian Cao, Ruming Geng, Cheng Yang, Shun Han, Simin Lei, Yong Li, Chunxu Wang

**Affiliations:** Research Institute for Special Steel Research, Central Iron and Steel Research Institute Company Limited, Beijing 100081, China; cyx726924@163.com (Y.C.); yc15599035705@163.com (C.Y.); hanshunfa@126.com (S.H.); leisimin@nercast.com (S.L.); liysea@163.com (Y.L.); wangchunxu@nercast.com (C.W.)

**Keywords:** energetic structural materials, high-entropy alloys, microstructures, mechanical properties

## Abstract

High-energy structural materials (ESMs) integrate a high energy density with rapid energy release, offering promising applications in advanced technologies. In this study, a novel dual-phase Ti_40_Zr_40_W_10_Mo_10_ high-entropy alloy (HEA) was synthesized and evaluated as a potential ESM. The alloy exhibited a body-centered cubic (BCC) matrix with Mo-W-rich BCC precipitates of varying sizes, which increased proportionally with the W content. The compressive mechanical properties were assessed across a range of strain rates, revealing that the W10 HEA sustained a compressive strength of 2300 MPa at a strain rate of 3000 s^−1^. This exceptional performance is attributed to the uniform distribution of circular Mo-W-rich BCC precipitates. Conversely, in the W13 HEA, the aggregated and large Mo-W-rich precipitates deteriorated its dynamic properties. Furthermore, deflagration behavior was observed during dynamic deformation of W10, highlighting its potential as a high-performance ESM.

## 1. Introduction

Energetic structural materials (ESMs) are vital for modern military and industrial applications due to their unique combination of exceptional mechanical properties and rapid energy release capabilities [1,2,3]. These advanced materials are engineered to endure extreme conditions while delivering substantial stored energy on demand, making them indispensable for applications requiring both high strength and controlled reactivity. Enhancing the mechanical performance and energy release efficiency of ESMs is critical for optimizing the destructive effectiveness of fragmentation warheads [4].

Generally, ESMs are typically categorized into two distinct types, composite and pure metallic forms, with the composite type encompassing variations such as metal–polymer [5], metal–metal oxide [6], and metal–metal mixtures [7]. While composite ESMs exhibit superior energy release efficiency, their lack of strong interphase bonds results in mechanical properties that are significantly inferior to those of traditional structural materials, rendering them unsuitable for use as load-bearing structural components. To address this, researchers have explored the use of amorphous materials [8], which are known for their high strength, to develop ESMs and address the mechanical shortcomings of conventional designs. However, amorphous alloys typically deform through localized shear bands, resulting in extremely poor plasticity and failing to meet structural performance requirements [9,10]. As a result, designing ESM compositions that combine high combustion enthalpy with superior mechanical properties has become a central focus of research worldwide.

The emergence of multi-principal high-entropy alloys (HEAs) has transformed the conventional alloy design, enabling the exploration of previously inaccessible regions within phase diagrams and unlocking unique material properties. This paradigm shift has catalyzed extensive research efforts in the field [11,12]. HEAs exhibit a range of exceptional characteristics compared to traditional alloys, including outstanding strength [13], radiation resistance [14], wear resistance [15], corrosion resistance [16], remarkable resistance to high-temperature softening [17], and distinctive physical properties. Recent studies have begun to investigate the potential of HEAs in the development of ESMs, opening new avenues for innovation in this domain.

In 2017, Bai et al. introduced and validated the concept of HEA ESMs, combining high strength, ductility, and chemical reactivity [18]. They observed a pronounced energy release and combustion phenomenon in the TiZrHfTa_0.53_ alloy during high-velocity penetration into a steel plate [18]. Subsequently, Dai et al. designed WMoFeNi HEA fragments that exhibit self-sharpening behavior and superior dynamic mechanical properties compared to conventional tungsten alloys [19]. This performance enhancement is attributed to the precipitation of an ultra-high-strength μ-phase during dynamic recrystallization, significantly improving their effectiveness in armor penetration and breaching applications. Additionally, the incorporation of tungsten into the TiZrHfTa_0.7_W_0.3_ HEA enhances its combustion potential, enabling substantial chemical energy release and improved energy delivery during high-velocity impact scenarios [3]. Recently, the TiZrMo multi-element alloy has garnered attention due to its exceptional yield strength [20], which is attributed to significant lattice distortion and substantial solid solution strengthening effects [21]. Furthermore, the propensity of Ti and Zr elements to readily combine with oxygen to produce a favorable energy release effect positions this alloy as a promising candidate for applications within the realm of ESMs [22,23].

In the present investigation, W was incorporated as a substitute for Mo in the TiZrMo HEA to elucidate its impact on the alloy’s microstructural evolution and mechanical behavior. Subsequently, the concentrations of Ti and Zr were augmented to engineer a novel dual-phase Ti_40_Zr_40_W_10_Mo_10_ HEA. This compositional modification is anticipated to enhance the energy dissipation efficiency of the alloy and to ameliorate its ductility by diminishing the valence electron concentration (VEC) [24]. The experimental findings indicate that the alloy possesses superior static and dynamic compressive characteristics, offering significant theoretical underpinnings for the development of HEA-based ESMs.

## 2. Materials and Methods

Ti-Zr-Mo-W ingots were prepared through a vacuum furnace using high-purity constituent elements. TiZrMo, Ti_33_Zr_33_Mo_25_W_8_, Ti_33_Zr_33_Mo_20_W_13_, Ti_33_Zr_33_Mo_15_W_18_, and Ti_40_Zr_40_Mo_10_W_10_ were determined as W_0_, W_8_, W_13_, W_18_, and W_10_, respectively. To achieve chemical homogeneity, given the substantial differences in density among the constituent elements, each ingot was subjected to a minimum of eight remelting cycles, with each cycle involving a thorough inversion of the ingots. The remelting temperature was set above the melting point of each constituent, particularly W, to facilitate a complete blend of the raw materials. Each remelting process included a minimum dwell time of 10 min in the liquid state to ensure uniformity throughout the material. Following the melting process, the specimens were drilled to obtain shavings, which were then analyzed for composition using chemical methods. The resulting composition is detailed in Table 1.

Phase constitution was characterized by a Rigaku X-ray diffraction (XRD) instrument (Rigaku Corporation, Tokyo, Japan) with Cu-Kα irradiation in the range of 20°~110° with a scanning rate of 5°/min. The composition and microstructure of the specimens were examined by transmission electronic microscopy (TEM) using a Tecnai-F20 microscope (Thermo Fisher Scientific, Portland, OR, USA) equipped with energy dispersive spectroscopy (EDS) under an accelerating voltage of 200 kV. For the TEM observation, specimens were mechanically ground to a 50 μm thickness and then twin-jet electropolished using H_2_SO_4_ (10%) and CH_4_O (90%) solution under −30 °C.

Compressive tests at ambient temperature were performed on an Instron machine (Instron Corporation, Norwood, MA, USA) at a strain rate of 0.001 s^−1^; the load accuracy of the equipment was ±0.4%, and the speed control accuracy was ±0.1%. And a non-contacting video extensometer was used to record the strain of materials, with the video resolution better than 0.06 μm. Each alloy with cylindrical samples with a diameter of 5 mm and a height of 6 mm was tested at least three times. The dynamic compressive mechanical properties at different strain rates were tested using a Split Hopkinson pressure bar (SHPB) apparatus (Changsha Baisen experimental equipment Co., Ltd., Changsha, China) with specimen dimensions of Φ5 mm × 6 mm; the strain rate of SHPB was 500–10,000 s^−1^. A high-speed camera (LUSTER lightTech Co., Ltd., Beijing, China) was used to record the progress of compression.

## 3. Results and Discussion

### 3.1. Phase Structure

**Figure 1:**
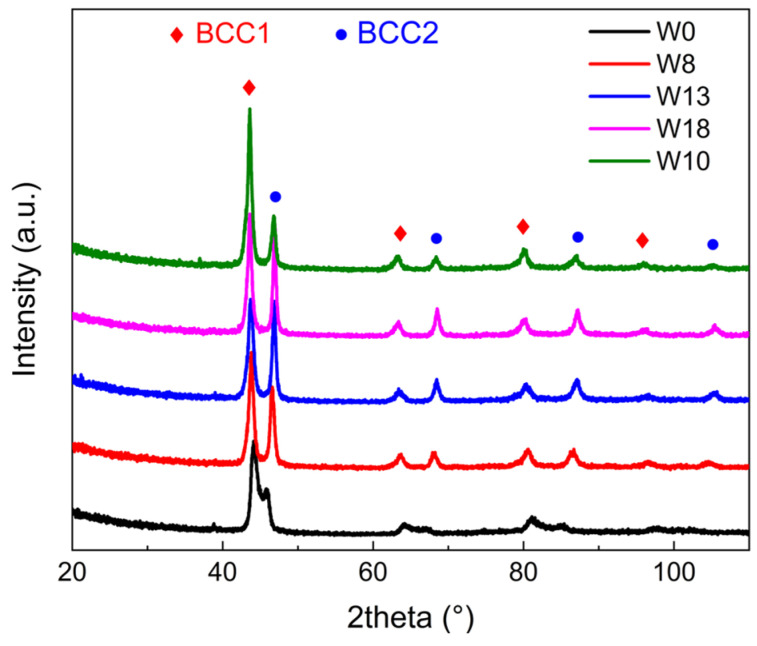
Room-temperature XRD patterns for HEAs with different W contents.

Figure 1 presents the XRD patterns of the HEAs with varying W contents, revealing diffraction peaks corresponding to two distinct body-centered cubic (BCC) phases. The lattice parameter, grain sizes, and contents of each BCC phase were calculated based on the positions, widths, and areas of the diffraction peaks, respectively, as summarized in Table 2. In all alloys examined, the content of the BCC2 phase is approximately 38%, except for the W10 alloy, indicating that substituting W for Mo does not significantly alter the phase content. However, the lattice parameters of the two phases are affected: the lattice parameter of the matrix phase (BCC1) increases with a higher W content, while that of the precipitated phase (BCC2) decreases. This suggests that W addition enhances the solid solution-strengthening effect of the matrix and increases lattice distortion.

In the W10 alloy, the increased Zr and Ti contents notably alter the phase proportions, reducing the BCC2 phase content to 23% and intensifying lattice distortion in both phases. These changes are likely to enhance the mechanical properties of the alloy.

### 3.2. Effect of W Element on Mechanical Properties and Microstructure

**Figure 2:**
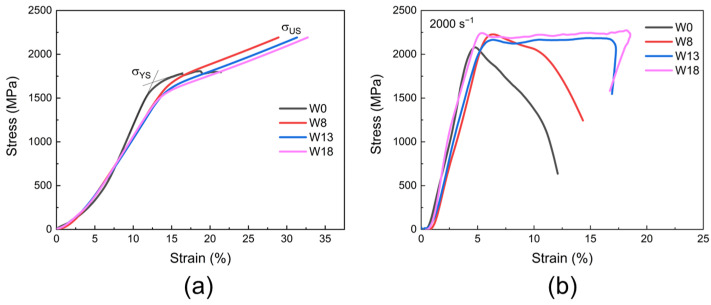
Effect of W content on mechanical properties: (**a**) quasi-static mechanical stress–strain compression curves; (**b**) dynamic mechanical stress–strain compression curves at 2000 s^−1^.

The quasi-static compressive stress–strain curves of the alloys with varying W contents are presented in Figure 2a. For the W0 alloy, the yield strength (*σ*_YS_) and the ultimate strength (*σ*_US_) are approximately 1620 MPa and 1810 MPa, respectively, with an elongation of ~22%. Serrated steps observed in the stress–strain curve of W0 suggest a susceptibility to plastic instability. The introduction of W into the alloy preserves the high yield strength while significantly improving both fracture strength and fracture strain [25]. For example, the W13 alloy demonstrates a yield strength of 1650 MPa, along with an ultimate strength of 2200 MPa and a fracture strain of 32%.

Figure 2b presents the dynamic engineering stress–strain curves of alloys with varying W contents, tested at high strain rates (2000 s^−1^) and at room temperature. Under dynamic conditions, the yield strength of each alloy is significantly enhanced compared with the quasi-static conditions. Notably, while the W0 and W8 alloys exhibit poor dynamic plasticity with elongations below 10%, the W13 and W18 alloys demonstrate superior dynamic mechanical properties, achieving ultra-high dynamic yield strengths (exceeding 2200 MPa) and uniform elongations of at least 17%. These results indicate that increasing the W content effectively enhances the dynamic mechanical performance of the alloy. And this improvement can be attributed to the high melting point and density of W, which mitigate the softening effects induced by temperature rise during high-speed deformation [17].

**Figure 3:**
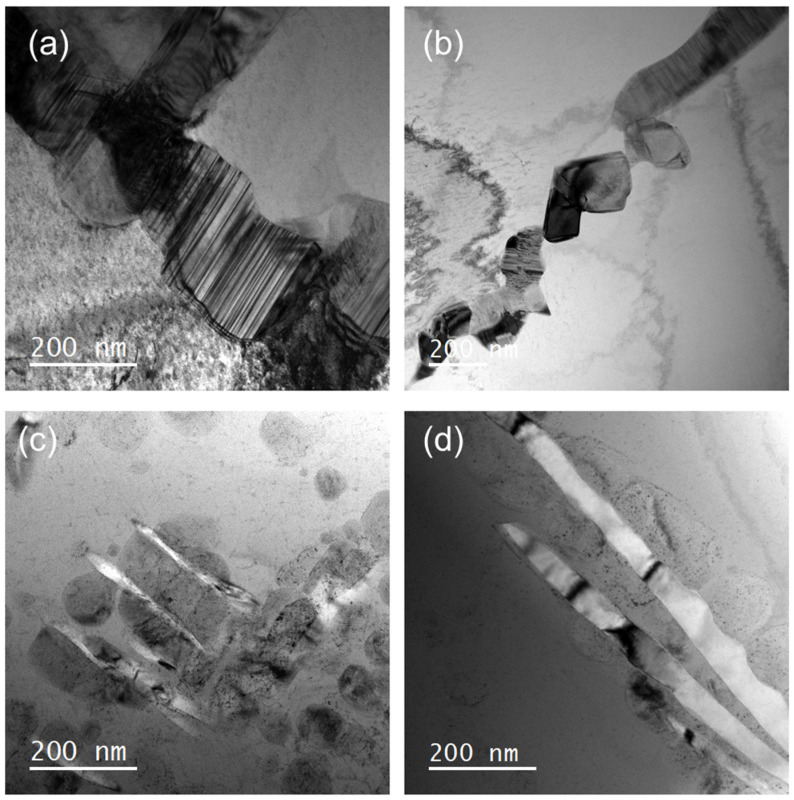
TEM images of alloys with different W contents: (**a**) W0; (**b**) W8; (**c**) W13; and (**d**) W18.

To elucidate the differences in mechanical properties of alloys with varying W contents, TEM characterization was conducted, as shown in Figure 3. The image reveals that the W0 alloy has a larger BCC2 phase above 200 nm, with a stacking fault structure (Figure 3a). This structure contributes to the high compressive yield strength of the W0 alloy but results in almost no plasticity. As W replaces Mo, the BCC2 phase undergoes significant changes, as seen in Figure 3b–d. The precipitated phase in the W8 alloy is only 100 nm with an irregular shape, as shown in Figure 3b. As the W content increases, the BCC2 phase transitions from an irregular shape to a disk-like morphology in the W13 alloy and further to an elongated form in the W18 alloy, accompanied with the gradual disappearance of stacking fault structures within the precipitate phase. These findings indicate that, for alloys with the same phase content, the shape and size of the BCC2 phase have a substantial impact on the dynamic mechanical properties.

**Figure 4:**
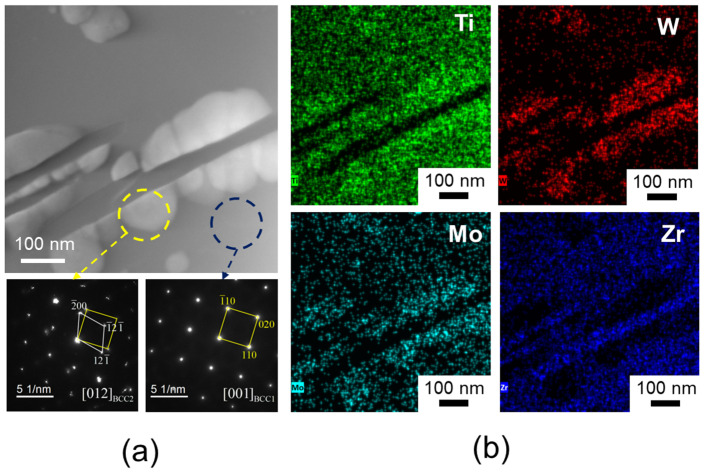
TEM images of W13 alloy: (**a**) bright-field image and SAED patterns; (**b**) EDS mappings.

To further investigate the influence of the two-phase structure on the alloy, the W13 alloy was selected for selected electron diffraction (SAED) and EDS analysis. The results are presented in Figure 4. Figure 4a displays the TEM morphology of the W13 alloy, showing that the BCC2 precipitates exhibit a disk-like shape embedded within the BCC1 matrix. This morphology differs from the dendritic structure reported for the W0 alloy in previous studies [20]. Additionally, while the BCC1 phase in both W13 and W0 alloys is Zr-rich [20], the BCC2 phase in the W13 alloy is enriched in W and Mo elements (Figure 4b). This composition results in smaller lattice parameters for the BCC2 precipitates in W13 compared to those in W0, which is likely attributable to the incorporation of W, an element with a smaller atomic radius, into the lattice structure, as confirmed by Table 2. Previous studies have demonstrated that W addition reduces the nucleation barrier of the BCC phase and decreases the valence electron concentration of HEAs, thereby promoting BCC phase nucleation [26]. Consequently, as the W content increases, the nucleation rate of the BCC2 precipitate is enhanced, refining the dendritic structure observed in the W0 alloy. This structural refinement significantly improves the mechanical properties of the alloy (Figure 2).

The results indicate that when the W content exceeds 8%, both the static and dynamic mechanical properties of the alloy are significantly enhanced. Therefore, the proportions of Ti and Zr were increased in the W10 alloy to achieve improved mechanical properties and enhanced energy performance.

### 3.3. Mechanical Properties of Ti_40_Zr_40_Mo_10_W_10_

**Figure 5:**
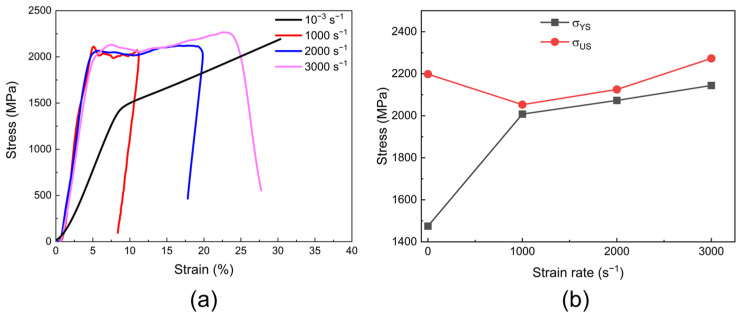
Compressive mechanical properties at various strain rates of W10 alloy: (**a**) stress–strain compression curves; (**b**) variations in yield strength (*σ*_YS_) and ultimate strength (*σ*_US_) with strain rate.

The engineering stress–strain curves of the W10 HEAs tested at room temperature under various strain rates (10^−3^, 1000, 2000, and 3000 s^−1^) are displayed in Figure 5a. Concurrently, the *σ*_YS_ and *σ*_US_ values for both alloys under different conditions are summarized in Figure 5b. Under static compression conditions, the W10 alloy demonstrates a yield strength of 1490 MPa, along with an ultimate strength of 2200 MPa and a fracture strain of 30%. Compared to quasi-static conditions, the dynamic yield strength of the W10 alloy increases significantly from 1490 MPa to 2100 MPa when the strain rate increases to 1000 s^−1^ (Figure 5a). And the W10 alloy achieves an ultimate strength of 2300 MPa at 3000 s^−1^. The change in ultimate strength with the strain rate is less than 10%, showing minimal strain rate dependence (Figure 5b). Notably, the W10 alloy demonstrates enhanced ductility at high strain rates, and at strain rates of 2000 s^−1^ and 3000 s^−1^, elongations of 20% and 24% were achieved, respectively (Figure 5a), which are significantly superior to the performance of other HEAs with varying W contents (Figure 2b). These results suggest that the dynamic mechanical properties of HEAs can be improved by optimizing the ratio of Ti and Zr elements at an optimal W concentration, with the W10 alloy serving as a prime example of achieving an excellent balance between strength and ductility under dynamic loading.

**Figure 6:**
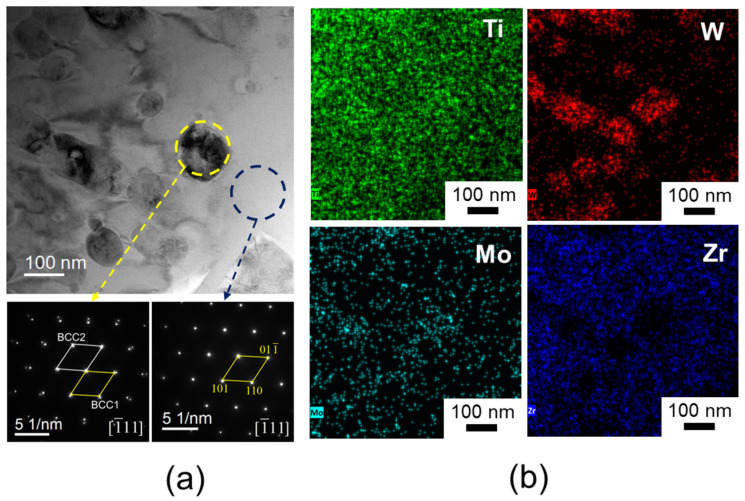
TEM images of W10 alloy: (**a**) bright-field image and SAED patterns; (**b**) EDS mappings.

The W10 and W13 alloys were further compared and analyzed; they are characterized by small changes in lattice parameters but large differences in the phase content. TEM techniques were employed to investigate the W10 alloy in detail, as shown in Figure 5. Figure 5a highlights the selected sample area, encompassing both the matrix and precipitates, along with their respective SAED patterns. The bright-field TEM image reveals spherical precipitates with diameters below 200 nm, contrasting with the matrix, and the size of these precipitate phases is significantly smaller than that of the W13 alloy (Figure 4a). A diffraction analysis confirms that the matrix corresponds to the BCC1 phase, while the precipitates correspond to the BCC2 phase, which is consistent with the XRD results (Figure 1). Elemental mapping (Figure 6b) indicates that the BCC2 phase is enriched with W and Mo, whereas the BCC1 matrix is rich in Zr, which is consistent with the image observed in W13 (Figure 4b). The uniform distribution of Ti suggests a relatively similar Ti content in both phases. Additionally, the elemental compositions of the BCC1 matrix and BCC2 precipitates in the W10 HEA are summarized in Table 3.

A microstructural analysis reveals that the differences in dynamic mechanical behavior between the W10 and W13 alloys can be attributed to variations in the size and distribution of the precipitates (Figure 4 and Figure 6). A microstructural examination indicates that the disparities in dynamic mechanical behavior between the W10 and W13 alloys are ascribed to the precipitate size and distribution. In the W10 HEA, the small, circular precipitates with uniform distribution impede dislocation slip during dynamic deformation, preventing the rapid expansion of adiabatic shear bands, thereby stabilizing plastic deformation and forestalling catastrophic failure. Furthermore, the smaller second-phase particles in the W10 alloy offer an increased number of interfaces for energy absorption, thereby enhancing toughness and overall dynamic mechanical properties [27]. In contrast, the larger precipitates in the W13 alloy fail to mitigate stress concentrations, which precipitates premature fracture.

**Figure 7:**
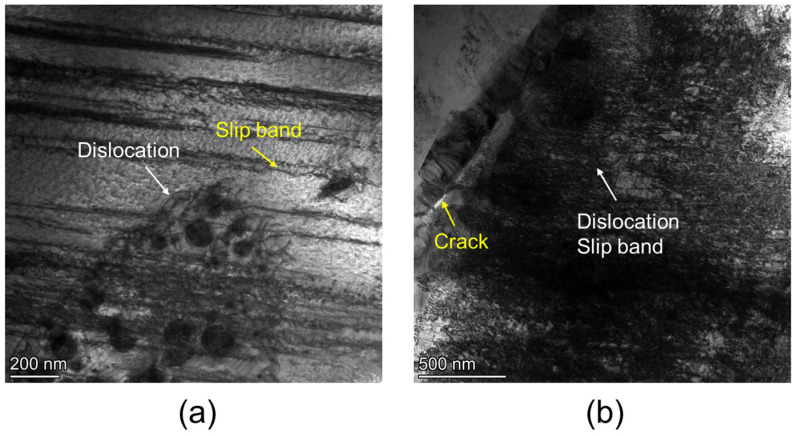
Bright-field TEM image after compression at strain rate of 2000 s^−1^: (**a**) W10 alloy; (**b**) W13 alloy.

To validate the above observations, the microstructures of the W10 and W13 alloys were further characterized following dynamic compression at a strain rate of 2000 s^−1^, as shown in Figure 7. Both alloys exhibited dislocation slip bands after dynamic compression. Notably, the fine BCC2 precipitates in the W10 alloy effectively impede dislocation motion without causing significant dislocation accumulation (Figure 7a). In contrast, the larger BCC2 precipitates in the W13 alloy lead to dislocation pile-up at the precipitate–matrix interface, resulting in crack formation (Figure 7b). These findings suggest that optimizing the Ti and Zr contents and carefully controlling the ratio of BCC1 to BCC2 phases can effectively enhance the mechanical properties of the alloy.

### 3.4. Energetic Characteristics of Ti_40_Zr_40_Mo_10_W_10_

**Figure 8:**
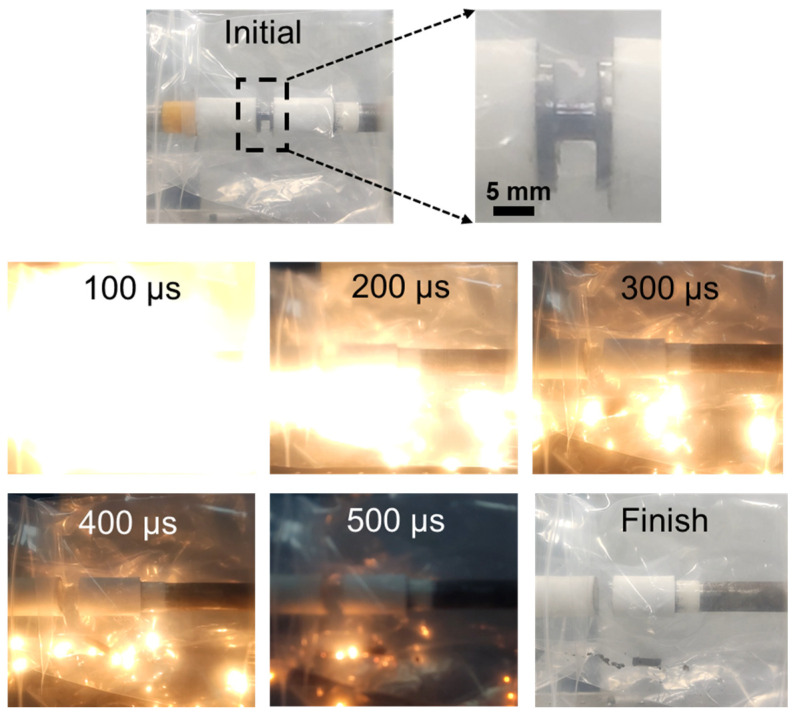
High-speed camera frames of the impact and reaction processes of the W10 HEA at 3000 s^−1^.

In order to better understand the dynamic deformation and the energy release process of the W10 HEA, a high-speed camera was used to capture the SHPB compression test processes, as seen in Figure 8. The initial frame shows the specimen before the test, with a clear view of the cylindrical shape and the dashed lines indicating the region of interest. As the test progresses, subsequent frames at 100 μs intervals reveal the onset of deformation, the propagation of the stress wave, and the increasing deformation of the specimen. At around 100 μs, incandescent light is observed in the investigated HEA, which could be explained by the ”hot spot” theory [28]. During compression, the specimen reacts violently with the air, and the internal accumulation of deformation energy and frictional heat lead to a sharp temperature increase, providing energy for “hot spots”. The frames at 200, 300, and 400 μs demonstrate intense deformation and the release of energy, characterized by the bright flashes and the significant change in the specimen’s shape. By 500 μs, the reaction nears completion, and the final frame shows the aftermath of the impact, with the specimen having undergone substantial deformation, and the energy release process is concluded. Sample fragments are distributed in the test chamber. The findings of this study substantiate that the W10 alloy possesses not only exceptional mechanical properties but also demonstrates superior strength and ductility during dynamic deformation. Moreover, it exhibits an outstanding energy release efficiency, making it a promising candidate for applications in energetic materials.

## 4. Conclusions

This study demonstrates that the addition of W significantly enhances the mechanical properties of TiZrMo alloys, with the phase ratio effectively being controlled by adjusting the elemental composition. As a result, a dual-phase Ti_40_Zr_40_Mo_10_W_10_ HEA was successfully fabricated and subjected to comprehensive quasi-static and dynamic loading experiments to evaluate its energy release capability and mechanical properties for potential structural applications. The Ti_40_Zr_40_Mo_10_W_10_ HEA demonstrated exceptional quasi-static mechanical performance, with a yield strength of 1490 MPa, an ultimate strength of 2200 MPa, and a fracture strain of 30%. Under dynamic loading at a strain rate of 3000 s^−1^, the alloy exhibited an even higher ultimate strength of 2300 MPa and maintained a fracture strain of 24%, showcasing its remarkable ability to retain plasticity under extreme conditions. Additionally, the Ti_40_Zr_40_Mo_10_W_10_ HEA displayed significant chemical energy release and a high energy release capacity. This combination of outstanding mechanical properties and energetic performance underscores the Ti_40_Zr_40_Mo_10_W_10_ HEA’s potential as a novel high-strength ESM. Importantly, future studies should explore the effects of incorporating additional principal elements, such as oxygen-affinitive elements like Hf, on the energetic properties of HEAs.

## Figures and Tables

**Table 1:** Chemical compositions of experimental alloys (wt.%).

Sample	W	Ti	Zr	Mo
W0	0	20.3	38.8	40.9
W8	18.0	18.6	35.4	28.0
W13	27.3	17.7	33.7	21.3
W18	35.8	16.9	32.1	15.2
W10	22.0	22.9	43.6	11.5

**Table 2:** Volume fractions, unit cell parameters, and grain sizes of two BCC phases in HEAs with different W contents.

Sample	BCC1		BCC2	
	Volume Fraction	a = b = c (Å)	Volume Fraction	a = b = c (Å)
W0	0.61	2.901	0.39	2.795
W8	0.63	2.918	0.37	2.755
W13	0.62	2.928	0.38	2.739
W18	0.61	2.932	0.39	2.737
W10	0.77	2.933	0.23	2.741

**Table 3:** The elemental contents of the matrix and precipitated phase in the W10 HEA.

Phases	Ti (at. %)	Zr (at. %)	Mo (at. %)	W (at. %)
BCC1	21.2	56.1	12.8	9.9
BCC2	21.4	14.7	28.8	35.1

## Data Availability

The original contributions presented in this study are included in the article; further inquiries can be directed to the corresponding author.
